# Hormone replacement therapy in women and risk of carpal tunnel syndrome: a systematic review and metaanalysis

**DOI:** 10.1186/s10195-023-00707-5

**Published:** 2023-06-12

**Authors:** Worapaka Manosroi, Pichitchai Atthakomol, Phichayut Phinyo, Pojsakorn Danpanichkul

**Affiliations:** 1 Division of Endocrinology, Department of Internal Medicine, Faculty of Medicine, Chiang Mai University, Chiang Mai, Thailand; 2 Clinical Epidemiology and Clinical Statistics Center, Faculty of Medicine, Chiang Mai University, Chiang Mai, Thailand; 3 Department of Orthopaedics, Faculty of Medicine, Chiang Mai University, 110 Intrawarorot Road Soi 2, Si Phum, Amphoe Mueang Chiang Mai, Chiang Mai, 50200 Thailand; 4 Department of Family Medicine, Faculty of Medicine, Chiang Mai University, Chiang Mai, Thailand; 5 Musculoskeletal Science and Translational Research (MSTR), Chiang Mai University, Chiang Mai, Thailand; 6 Department of Microbiology, Faculty of Medicine, Chiang Mai University, Chiang Mai, Thailand

**Keywords:** Hormone replacement therapy, Estrogen, Progesterone, Carpal tunnel syndrome

## Abstract

**Background:**

Carpal tunnel syndrome (CTS) is the most common entrapment mononeuropathy. Menopausal status and/or estrogen level may play a role in CTS. The evidence regarding the association between hormone replacement therapy (HRT) in postmenopausal women and CTS is still conflicting. This meta-analysis aimed to investigate the association between carpal tunnel syndrome (CTS) and women using hormone replacement therapy (HRT).

**Methods:**

A search was conducted in the PubMed/Medline, Scopus, Embase, and Cochrane databases, from their inception through July 2022. Studies which reported on the association between any type of HRT use and the risk of developing CTS in postmenopausal women compared to a control group were included. Studies which did not include a control group were excluded. Of the 1573 articles extracted from database searches, seven studies involving 270,764 women were included of which 10,746 had CTS. The association between CTS and HRT use was evaluated using the pooled odds ratio (OR) with a 95% confidence interval (CI) under random-effects modelling. Risk of bias in each study was assessed using the Newcastle–Ottawa Scale (NOS) and version 2 of the Cochrane tool for assessing risk of bias in randomized trials (RoB 2).

**Results:**

HRT use showed no statistically significant association with a higher risk of CTS with pooled odds ratio (OR) 1.49, 95% confidence interval (CI) 0.99–2.23, and *p* = 0.06, although high heterogeneity among the studies was observed (*I*^2^ 97.0%, *Q*-test *p*-value < 0.001). Subgroup analysis of groups in non-randomized controlled studies showed a significantly increased risk of CTS, while groups in randomized controlled studies showed a decreased risk of CTS (pooled OR 1.87, 95% CI 1.24–2.83 versus pooled OR 0.79, 95% CI 0.69–0.92, respectively) with the *p*-value of group difference < 0.001. The risk of bias in most of the included studies was estimated to be low.

**Conclusions:**

This meta-analysis supports the safety of using HRT in postmenopausal women with potential risk factors for CTS.

**Level of evidence:**

I, Prognosis.

*Registration*: INPLASY (202280018).

**Supplementary Information:**

The online version contains supplementary material available at 10.1186/s10195-023-00707-5.

## Introduction

Carpal tunnel syndrome (CTS) is one of the most common causes of hand disability leading to inability to perform some tasks or loss of work [1]. The incidence of CTS is ~ 2.3 cases per 100 person-years in the general population [2]. The peak age of onset is around 40–60 years and is ten times more common in females than in males [3].

The etiology of CTS has been described as multifactorial, including genetic predisposition, a history of repetitive wrist movements, obesity, autoimmune disorders, and pregnancy [1]. As mentioned above, CTS is more commonly observed in females and the incidence increases with age. For those reasons, it has been generally presumed that menopausal status and/or estrogen level may play a role as one of the etiologies of CTS. Aromatase inhibitors, which can lower serum estrogen in breast cancer patients, has been demonstrated to increase the incidence of CTS by triggering inflammation and edema in the flexor compartment of the wrist [4[. In addition, estrogen receptors have been found to be present in transverse carpal ligament (TCL) and synovial tissue in CTS patients [5]. Taken together, this evidence suggests that estrogen may have a role in the pathogenesis of CTS.

Recent evidence regarding the association between hormone replacement therapy (HRT) in postmenopausal women and CTS is still conflicting [6, 7]. HRT is usually prescribed for postmenopausal women with vasomotor symptoms. Estrogen is the major component of HRT preparations. Progesterone is also prescribed to prevent endometrial hyperplasia, except in women who have undergone a hysterectomy. A secondary analysis of a large randomized controlled trial demonstrated a protective effect of HRT on the incidence of CTS in postmenopausal women [7]. In contrast, a large nationwide population-based study of women in Taiwan reported increased risk of CTS with HRT [6]. Based on these contradictory results, we aimed to perform a systematic review and metaanalysis to clarify the association between CTS and women using HRT.

## Methods

This study followed the Preferred Reporting Items for Systematic Reviews and Meta-analyses (PRISMA) guidelines [8]. The protocol was registered with INPLASY.

### Searches

A comprehensive search in PubMed/Medline, Scopus, Embase, and Cochrane databases was conducted from their inception through July 2022. Included keywords were “estrogen replacement therapy OR hormone replacement therapy OR estrogen OR hormones OR postmenopausal women” AND “carpal tunnel syndrome”. Full details of keywords are provided in the Additional file 1: Appendix.

Two authors conducted the searches and independently screened each publication for titles and abstracts. Relevant studies were extracted and underwent screening of the full text for inclusion criteria. Then the assessment of methodological quality of the included studies and data extraction were performed separately by two authors who also conducted the data extraction. Any inconsistencies were discussed with a third author and resolved through consensus.

### Study inclusion and exclusion criteria

Inclusion criteria were: (1) cross sectional studies, case–control stuies, or randomized controlled trials, which reported the association of any type of HRT used in postmenopausal women and the risk of developing CTS compared with a control group, (2) CTS diagnosed by medical records, International Classification of Diseases (ICD) or Current Procedural Terminology (CPT) code, clinical diagnosis or other measures (ultrasonography or electrodiagnosis), and (3) the risk was reported as either an adjusted or unadjusted odds ratio (OR) or as a hazard ratio (HR). Exclusion criteria were studies without a control group, those published in a language other than English, review articles, case reports, abstracts, and animal studies.

Manual searches were also conducted to identify references cited in included studies as well as in non-included reviews.

### Data extraction strategy

The process of data extraction was independently executed by two authors. The variables extracted from each study included: (1) study characteristics, i.e., the name of the first author, year of publication, country where the study was conducted and study design, (2) number of patients with CTS and without CTS, (3) number of patients who used HRT and who did not use HRT, (4) patient characteristics, i.e., means and standard deviations (SD) of age, percentage of males, mean and SD of the duration of HRT use, if available, (5) OR or HR with 95% confidence interval (CI) of the risk of developing CTS in women who used HRT, and (6) whether the reported OR or HR were adjusted for confounders. Studies which presented the risk of developing CTS as HR were manually converted to OR using the number of reported events in the HRT-exposed and the HRT non-exposed groups. There was one study which contained two cohorts, each of which were administered different preparations of HRT [conjugated equine estrogen (CEE) and estrogen plus progesterone (E + P)] [7]. The OR obtained from each of the cohorts was individually analyzed.

### Study quality assessment

The Newcastle–Ottawa Scale (NOS) for case–control studies was used to assess the risk of bias in case–control and cross-sectional studies. Version 2 of the Cochrane tool for evaluating the risk of bias in randomized trials (RoB 2) was employed to determine the risk of bias in randomized controlled trials [9]. Two authors independently assessed the risk of bias. Inconsistencies were then clarified through discussion with the third author.

### Data synthesis and presentation

Meta-analysis was performed using the STATA program version 17.0 (StataCorp LLC, College Station, TX, USA). Pooled ORs were calculated using the logarithm of effect size and standard error from each study. Pooled OR was calculated using random effect modelling using the method of restricted maximum likelihood (REML) due to rare binary outcomes and large differences in study size. This random effect model method has been recommended over other methods for estimating heterogeneity variance [10]. The statistical significance level for this metaanalysis was set at *p* < 0.05.

### Potential effect modifiers and reasons for heterogeneity

To evaluate the statistical heterogeneity among the studies, the I^2^ statistic was assessed. *I*^2^ values > 75% with a significant Cochran *Q* test (*p* < 0.05) were considered to indicate high heterogeneity. *I*^2^ values < 75% were regarded as moderate to high heterogeneity. Publication bias was evaluated by funnel plots and Egger’s linear regression tests. Funnel plots should be a symmetrical inverted funnel when there is an absence of publication bias and asymmetrical when there is publication bias. A *p*-value of < 0.05 was considered an indication of statistically significant publication bias in Egger’s regressions.

As there were differences in study design among the studies, subgroup analysis categorized by randomized controlled trial studies and non-randomized controlled trial studies were performed. In addition, both adjusted and unadjusted ORs were reported for the included studies. Subgroup analysis of whether the reported OR was adjusted for confounders or not was also conducted. Finally, an analysis of a study which identified the incidence of CTS by retrieving data based on CTS releasing procedure was conducted [11]. Sensitivity analysis was also performed by removing that study from the analysis.

### Certainty assessment

Independent grading of quality of evidence was performed by two authors using the Grading of Recommendation, Assessment Development and Evaluation (GRADE) tool. The grading procedure, described elsewhere in this study, followed Schünemann et al. [12]. Any discrepancies were resolved by the third author.

## Results

### Review statistics

**Fig. 1:**
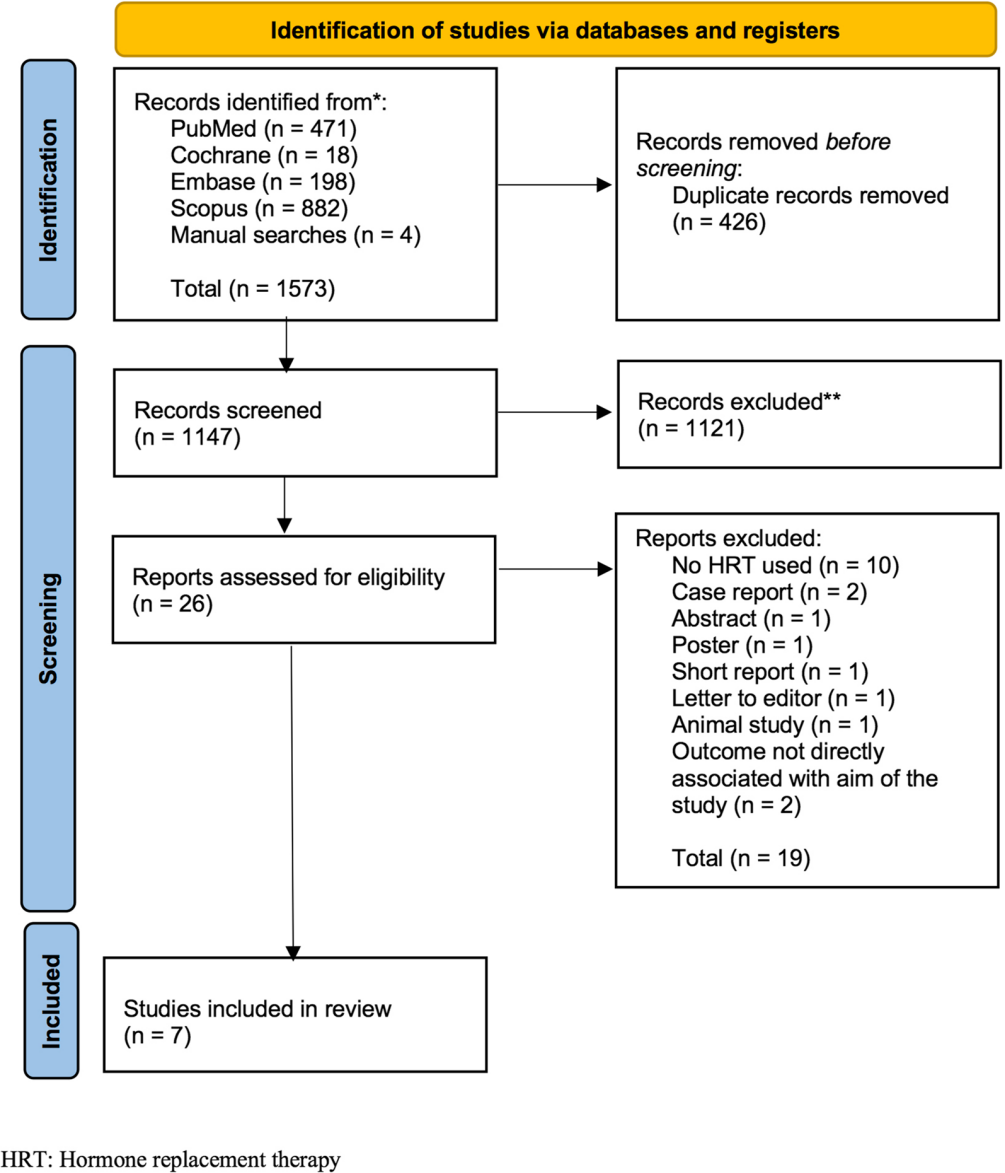
Prisma flow diagram

Of the 1573 articles extracted from database searches, Scopus yielded 882, PubMed yielded 471, Embase yielded 198, Cochrane yielded 18, and 4 were found from a manual search. From the extracted articles, 426 duplicates were eliminated. A screening of the remaining 1147 titles and abstracts resulted in exclusion of a further 1121 articles, which were not pertinent to the study objective. The full texts of the remaining 26 articles were collected and reviewed, resulting in the exclusion of an additional 19 articles owing to various reasons. Finally, a total of 7 studies were included [6, 7, 11, 13–16]. The PRISMA selection process used is shown in Fig. 1.

### Study characteristics

Characteristics of the seven included studies are shown in Table 1. One of the included studies was a randomized controlled trial 7, five were case–control studies, and one was a nested case–control with 1:1 matching [6, 11, 13, 15, 16] and one was a cross-sectional study^14^. One randomized trial by Rousan et al. included two cohorts which used two different regimens of HRT: CEE and E + P [7]. One study diagnosed CTS by ultrasonography or electrodiagnosis [14], while the others diagnosed CTS by either ICD or CPT code or by medical records. One study reported the risk of developing CTS as HR, which was converted to OR as mentioned above. The incidence of CTS among the studies ranged from 3.8 to 20%.

**Table 1:** Baseline characteristics of the included studies

Author, year	Country	Study design	Total population	Carpal tunnel syndrome, *n* (%)	HRT used in case, *n* (%)	HRT used in control, *n* (%)	Age (mean ± SD)	Duration of HRT used, year (median, IQR)	Type of HRT	Adjusted data
Tang, 2022 [6]	Taiwan	Case–control	118,309	4535 (3.8)	2,334 (51.5)	31,958 (28.1)	51	N/A	N/A	Yes
Rousan, 2018 [7]	USA	Randomized-control trial	16,053	841 (5.2)	376 (44.7)	7704 (50.6)	65.9 ± 5.6	–	–	No
CEE trial			6,833	465 (6.8)	203 (43.6)	3177 (49.8)	65.7 ± 6	7.2 (6.4, 8.1)	CEE	No
EP trial			9,220	376 (4.1)	173 (46)	4527 (51.2)	66.2 ± 5.4	5.6 (4.8, 6.5)	EP	Yes
Ricco, 2016 [14]	Italy	Cross sectional	631	48 (7.6)	39 (81.2)	264 (45.2)	38.1 ± 7.8	> 5	N/A	Yes
Geoghegan, 2004 [16]	UK	Case–control	16,955	3391 (20)	556 (16.4)	1799 (13.3)	46 ± 20	N/A	N/A	Yes
Ferry, 2000 [15]	England	Nested case–control	2528	1264 (50)	61 (4.8)	47 (3.7)	41.9	N/A	N/A	No
Solomon, 1999 [11]	USA	Case–control	115,205	627 (16.7)	5 (0.7)	3 (0.8)	73% over 64 years old	N/A	N/A	Yes
Dieck, 1985 [13]	USA	Case–control	1083	40 (3.8)	21 (52.5)	329 (31.5)	Range 45–74 years old	N/A	N/A	No

*HRT* hormone replacement therapy, *N/A* not available, *SD* standard deviation, *IQR* interquartile range, *CEE* conjugated equine estrogen, *EP* estrogen and progestin

### Study quality assessment

**Fig. 2:**
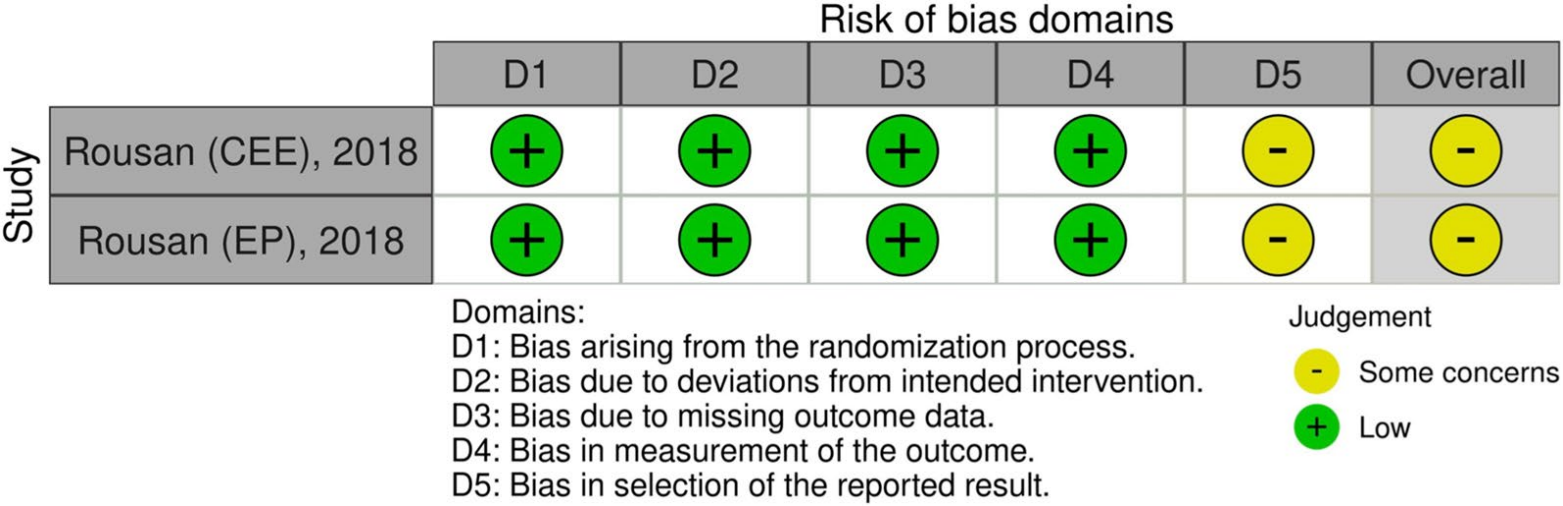
Risk of bias assessment by RoB 2 in the randomized controlled study

Evaluating by NOS, only one study showed moderate risk of bias (NOS score = 6) due to unclear diagnosis of CTS (ICD code). In that study, the control group was derived from hospital control and adjustment was made for only one confounder. The other six studies demonstrated a low risk of bias with NOS scores ≥ 7 (Table 2). By using the RoB 2 assessment tool for randomized controlled trials, some bias concerns were observed on the basis of the outcomes derived from secondary analysis of the studies (Fig. 2).

**Table 2:** Risk of bias in each study evaluated by Newcastle–Ottawa Scale (NOS)

First author, year	Selection				Comparability	Exposure			Total
	Is the case definition adequate	Representativeness of the cases	Selection of controls	Definition of controls		Ascertainment of exposure	Same method of ascertainment for cases and controls	Non-response rate	
Tang, 2022 [6]		*		*	**	*	*	*	*******
Ricco, 2016 [14]	*	*	*	*	**		*	*	********
Geoghegan, 2004 [16]		*		*	**	*	*	*	*******
Ferry, 2000 [15]		*		*	**	*	*	*	**** ***
Solomon, 1999 [11]		*		*	**	*	*	*	*******
Dieck, 1985 [13]		*		*	*	*	*	*	**** **

### Quantitative synthesis/metaanalysis

**Fig. 3:**
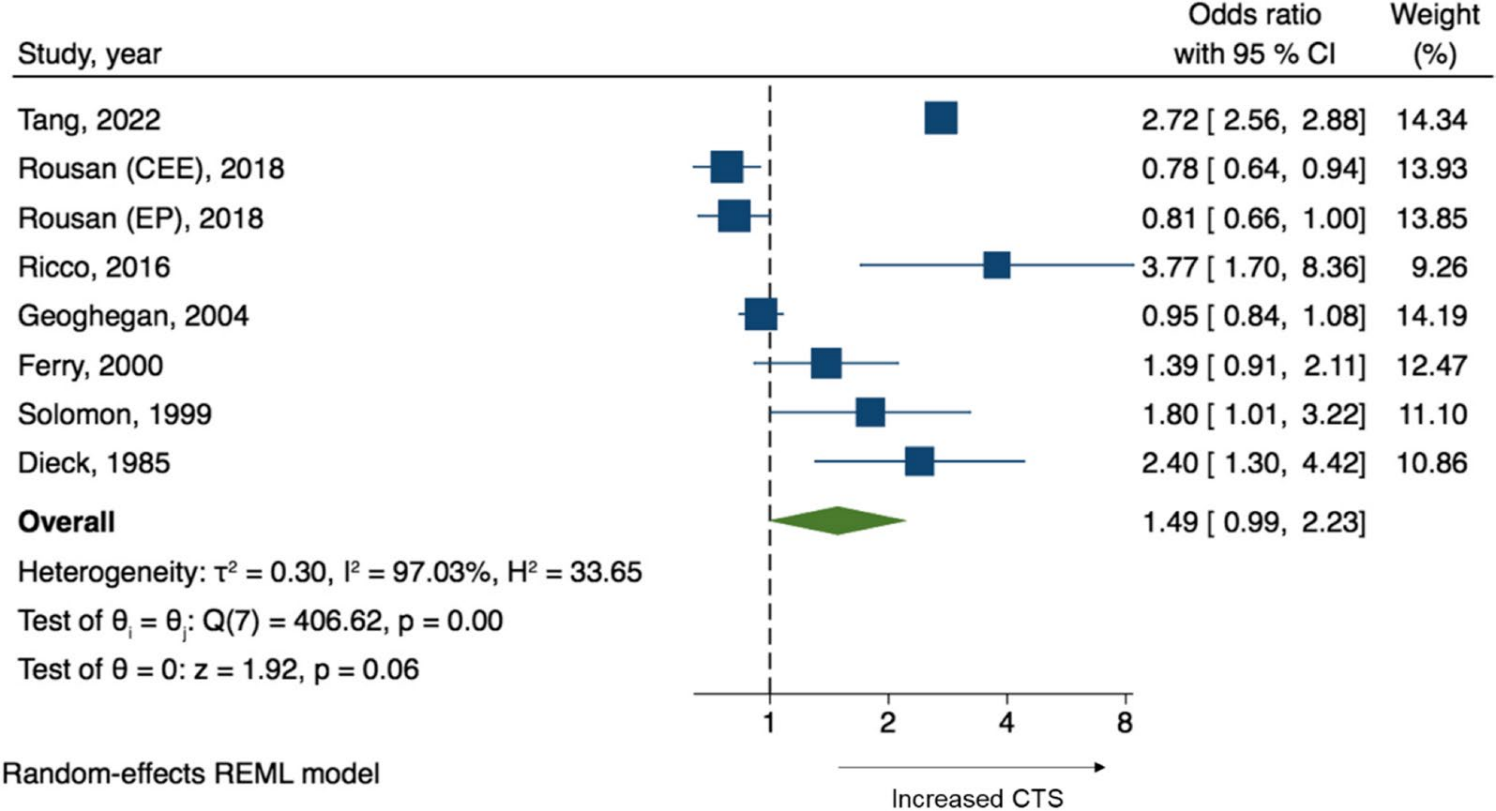
Forest plots of the odds ratio of carpal tunnel syndrome in women using HRT and women not using HRT

Seven studies with a total of 270,764 patients were included in this metaanalysis. A total of 10,746 patients had CTS. Overall, HRT use showed no statistically significant association with increased risk of CTS (pooled OR 1.49, 95% CI 0.99–2.23; *p* = 0.06; *I*^2^ 97.0%, *Q*-test *p*-value < 0.001) as shown in Fig. 3.

**Fig. 4:**
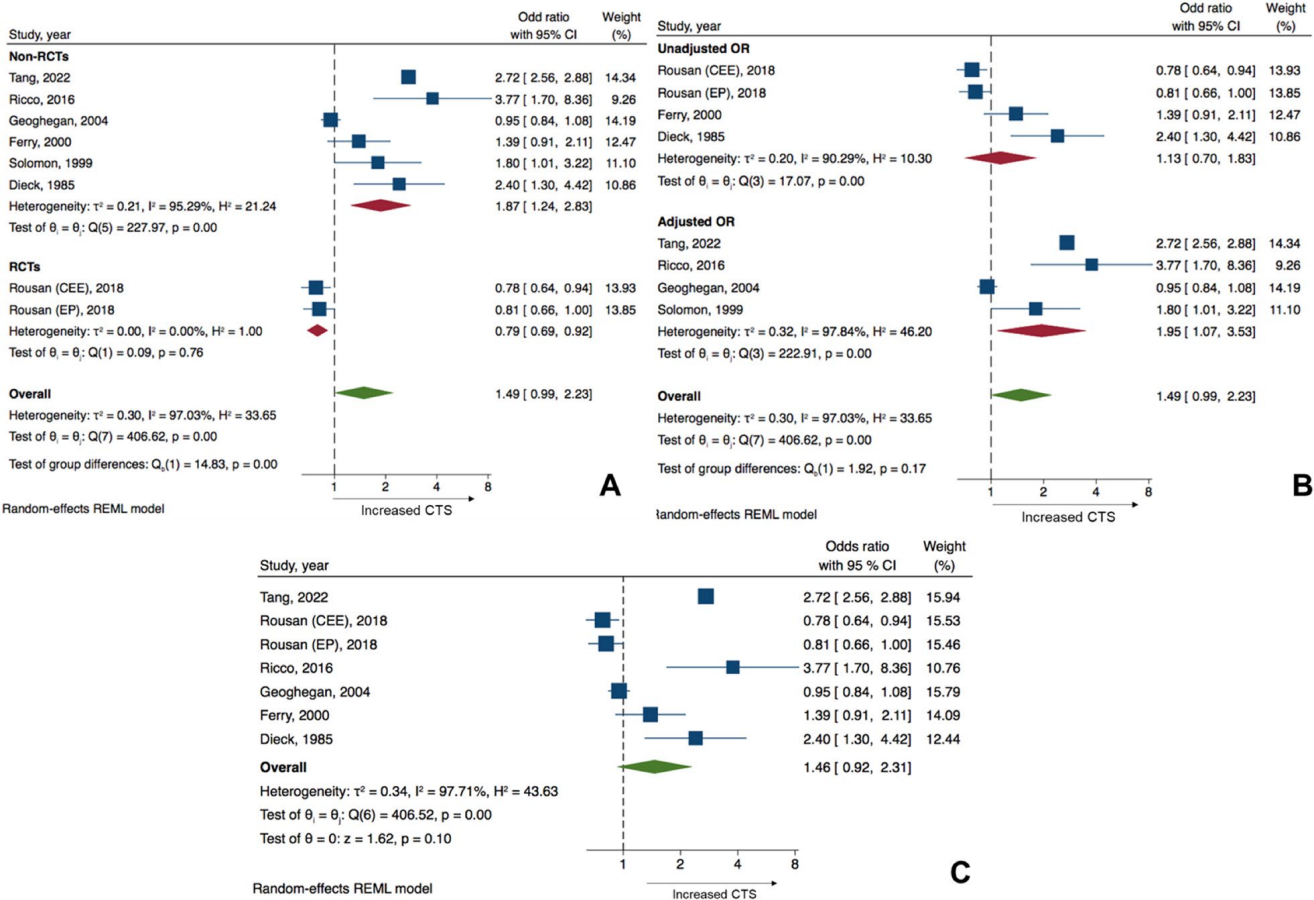
Subgroup analysis of odds ratio of carpal tunnel syndrome between women using HRT and women not using HRT categorized by (**A**) study design, (**B**) adjusted or unadjusted for confounders of reported OR, and (**C**) sensitivity analysis removing studies which identified the incidence of CTS by retrieving data from documentation of CTS release procedures

Regarding subgroup analysis, after categorizing by study design, significant group differences were observed among the subgroups with *p* < 0.001. In the subgroups in the randomized controlled study, which contained two cohorts, HRT use showed a significant decreased risk of CTS (pooled OR 0.79, 95% CI 0.69–0.92, *I*^2^ 0%, *Q*-test *p*-value 0.76) (Fig. 4A). In subgroups with non-randomized controlled studies, significantly increased risk of CTS in women using HRT was demonstrated (pooled OR 1.87, 95% CI 1.24–2.83, *I*^2^ 95.3%, *Q*-test *p-*value < 0.001). There was a reduction in heterogeneity only in the subgroup with randomized controlled trials. Regarding subgroup analysis by adjusted or unadjusted OR, only the subgroups with adjusted OR showed significant increased risk of CTS in women who used HRT (OR 1.95, 95% CI 1.07–3.53, *I*^2^ 97.8%, *Q*-test *p*-value < 0.001). Subgroups which reported unadjusted OR showed non-significant association between HRT and the occurrence of CTS, which was comparable to the primary analysis (OR 1.13, 95% CI 0.70–1.83, *I*^2^ 90.3%, *Q*-test *p*-value < 0.001). However, there was no significant group difference in this subgroup, with a *p* value of 0.17. There was no improvement in heterogeneity among the studies in this subgroup (Fig. 4B).

Results of sensitivity analysis after omitting the study which identified the incidence of CTS on the basis of CTS releasing procedure revealed comparable OR with the primary analysis (pooled OR 1.46, 95% CI 0.92–2.31). This sensitivity analysis also showed non-significant results, which is comparable to the primary analysis with *p* = 0.10 and the heterogeneity among the studies remained the same (*I*^2^ 97.7%, *Q*-test *p*-value < 0.001) (Fig. 4C).

### Reporting biases

**Fig. 5:**
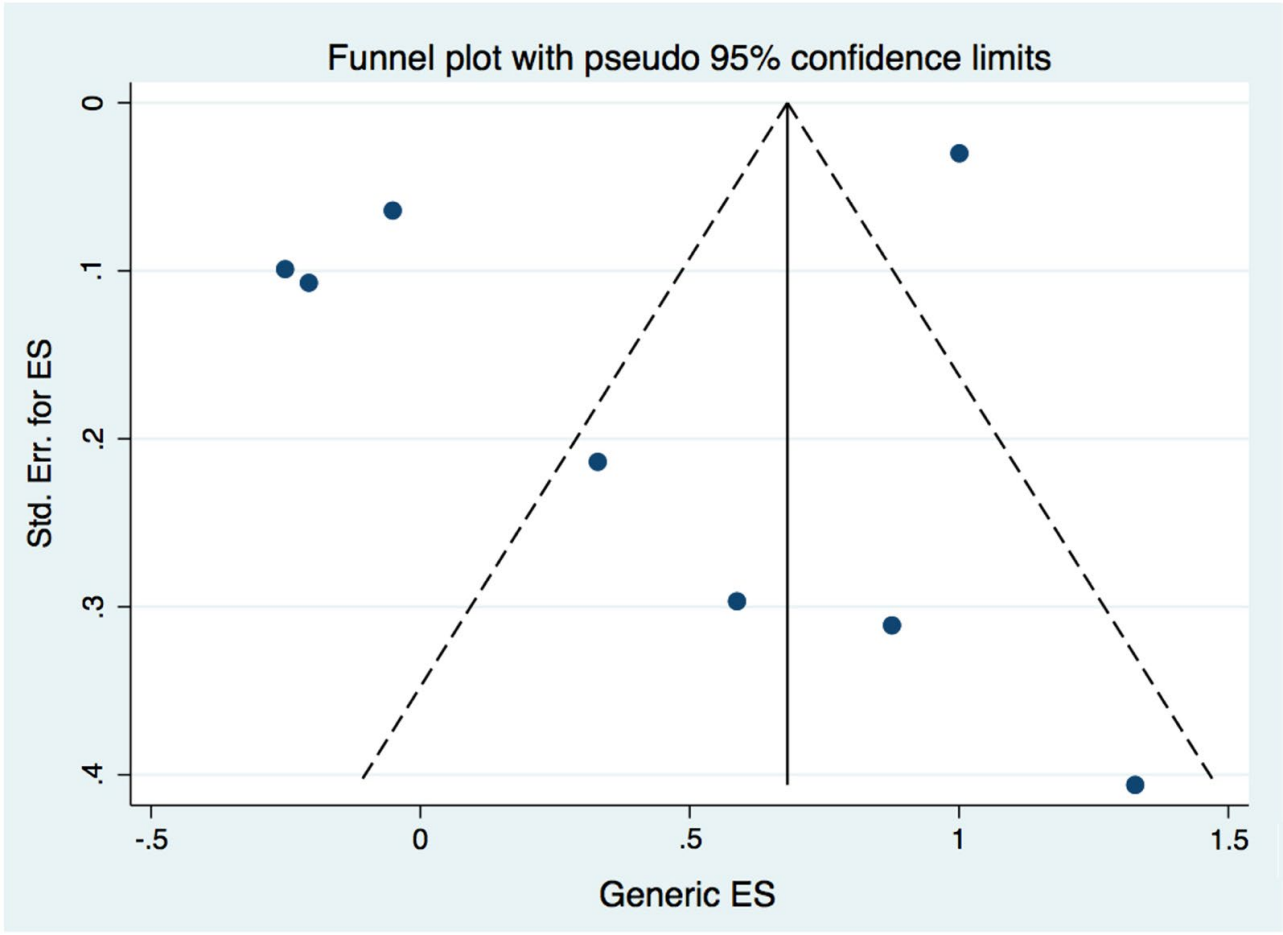
Funnel plot of seven included studies

Egger’s regression test found no evidence of publication bias with a *p*-value of 0.244. Similarly, the funnel plot was symmetrical (Fig. 5).

### Evidence of effectiveness

According to the GRADE assessment for certainty of the evidence, most of the reported risk were obtained from observational studies, which had a low rating for quality of evidence. No serious risk of bias, imprecision, indirectness, or publication bias was found in the synthesized metaanalysis. However, there was grading downward due to inconsistency of effect (high heterogeneity). Therefore, the summary certainty of evidence was very low.

## Discussion

This systematic review and meta-analysis is the first to examine the association between HRT and CTS. We found that women using HRT demonstrated a non-significant relationship with increased risk of CTS.

The link between estrogen, a sex steroid, and CTS has been reported in both human and in vitro studies. One study suggested that TCL was a target tissue for estrogen action as TCL in CTS patients expresses higher levels of estrogen receptor (ER) than synovial tissue does. ER expression reaches its peak in women aged 50–70 years (postmenopausal stage), which correlates with the age group that has a high incidence of CTS [5]. Increased expression of ER can lead to fibroblast proliferation and collagen synthesis at TCLs [17]. Another plausible mechanism is that high ER expression can induce immune activation and increase proinflammatory cytokines, resulting in synovitis and synovial hyperplasia [18]. When these changes occur in a carpal tunnel, they might be a factor in the development of CTS.

Indirect evidence of the association of estrogen with CTS has been reported. A high incidence of CTS has been reported in pregnant women, especially during the third trimester when it reaches ~ 62% [19]. One of the important predisposing factors for CTS in pregnant women is the fluctuation of estrogen levels. Other factors are fluid accumulation with a tendency to edemas, nerve hypersensitivity and glucose level fluctuations [20]. A rare event of CTS has been reported in patients treated with an aromatase inhibitor, a form of endocrine therapy, which blocks the conversion of androgen to estrogen [21]. Another interesting study reported that women undergoing bilateral oophorectomy had a higher incidence of CTS than normal women [22]. Regarding the use of oral contraceptive pills (OCP), the results were inconclusive, with some data suggesting a positive association with CTS [23]. However, recent data have reported that the new generation of OCP, which contains progesterone with anti-mineralocorticoid activity, has a protective effect against CTS by decreasing the severity of volume retention [24]. All this indirect evidence could be an indication that estrogen may play a crucial role in the pathogenesis of idiopathic CTS.

Multiple human studies of the relationship of HRT and CTS have been conducted, although the results are conflicting. For example, a large randomized controlled study, which reported the occurrence of CTS as a secondary outcome in a WHI cohort, showed a protective effect of HRT in postmenopausal women on the incidence of CTS [7]. In contrast, a large population-based case–control study found that, after adjusting for confounding factors related to CTS (age, diabetes, rheumatoid arthritis, hypothyroidism, gout, and obesity), HRT increased the risk of CTS by a factor of 2.7 times [6]. Both of these studies were included in our metaanalysis. There have been multiple theories that attempt to explain the increased incidence of CTS when HRT is used. For example, HRT can upregulate ER receptors in TCL, which can then lead to an increase in fibroblast and synovial lining cells, thus causing CTS [5]. Another theory is that decreased estrogen levels per se in postmenopausal women, a population which commonly uses HRT, can lead to high levels of inflammatory cytokines. These cytokines may contribute to cellular proliferation, angiogenesis, edematous changes of synovial tissue, finally resulting in CTS [25]. In contrast, another explanation for the protective effect of HRT is that during menopause, there is an increase in fat content at the wrist area, which might respond favorably to HRT and thus reduce the risk of CTS [26]. Additionally, some estrogen preparations have been found to have a positive effect on prostaglandin E2 and other inflammatory markers. This mechanism leads to the reduction of tenosynovitis, which is one of the pathophysiologies of CTS [27]. Nevertheless, the pathogenesis of the association between HRT and CTS is still inconclusive and needs further study.

In terms of subgroup and sensitivity analysis, only subgroup analysis stratified by either randomized or non-randomized studies has shown significantly different results between groups. This could be explained by differences in study design. A randomized controlled trial has been reported to be the best tool for examining the cause-and-effect relationship between intervention and outcome because it could minimize allocation bias and reduce confounding factors [28]. Randomized studies have provided more specific details in terms of types (CEE, E + P) and duration (7.2 and 5.6 years) of HRT use, while data on the types and duration of HRT used in non-RCTs were not available (Table 1). Differences in types and duration of HRT use could potentially affect the results in different groups. Further study using large randomized controlled methods should be performed to address this currently inconclusive issue. In addition, there was one study that diagnosed CTS by retrieving the data from documentation of the CTS releasing procedure. Sensitivity analysis by removing this study was performed due to, to diagnose CTS based on CTS release procedure, only cases with high severity of diseases were included, which can lead to selection bias in the study. However, the sensitivity analysis showed no different result with primary analysis.

The strengths of this first meta-analysis to address the association of HRT and CTS include, first, that the majority of the included studies had a low risk of bias, and second, subgroup and sensitivity analyses were conducted to identify the source of heterogeneity, and third, no publication bias was found among the studies.

We acknowledge some limitations in this meta-analysis. First, only one randomized controlled trial was included resulting in a high level of heterogeneity among the studies. Second, the included studies used a variety of measures with different diagnostic accuracy to diagnose CTS, resulting in a very wide range of incidence of CTS among the studies. Third, the details of the HRT used in the majority of the included studies were unclear e.g., type of HRT preparation and duration of use were not documented.

No significantly increased risk of CTS was demonstrated in women who used HRT. This meta-analysis supports the safety of using HRT in postmenopausal women who have underlying risk factors for the development of CTS, e.g., hypothyroidism, diabetes mellitus, and obesity. Future large randomized controlled trials should be conducted to provide confirmation of this result.

## Supplementary Information

**Additional file 1: Appendix **shows included keywords in comprehensive search

## Data Availability

The datasets used and/or analyzed during the current study are available from the corresponding author on reasonable request.

## Abbreviations

CEE: Conjugated equine estrogen
CI: Confidence interval
CPT: Current procedural terminology
CTS: Carpal tunnel syndrome
ER: Estrogen receptor
E + P: Estrogen plus progesterone
GRADE: Grading of Recommendation, Assessment Development and Evaluation
HR: Hazard ratio
HRT: Hormone replacement therapy
ICD: International Classification of Diseases
NOS: Newcastle–Ottawa Scale
OCP: Oral contraceptive pills
OR: Odds ratio
PRISMA: Preferred Reporting Items for Systematic Reviews and Meta-analyses
REML: Restricted maximum likelihood
RoB: Risk of bias
TCL: Transverse carpal ligament
