# Supplementary material for: Hormone replacement therapy in women and risk of carpal tunnel syndrome: a systematic review and metaanalysis
Source: J Orthop Traumatol. 2023 Jun 12;24:26. doi: 10.1186/s10195-023-00707-5 (PMC10260719; doi:10.1186/s10195-023-00707-5)
Supplement: Supplementary file 1 — Additional file 1: Appendix shows included keywords in comprehensive search [file 10195_2023_707_MOESM1_ESM.docx]

Additional file 1

Appendix shows included keywords in comprehensive search.

| Databases | Keywords |
| --- | --- |
| PubMed | ("Estrogen Replacement Therapy"[Mesh] OR "hormonal replacement therapy" OR “estrogen” OR "hormones" OR "postmenopausal" OR "risk factor") AND "Carpal Tunnel Syndrome"[Mesh] |
| Embase | 'carpal tunnel syndrome'/exp AND ('estrogen therapy'/exp OR 'postmenopause'/exp OR 'hormone'/exp OR 'estrogen'/exp) |
| Scopus | TITLE-ABS-KEY ( ( "Estrogen Replacement Therapy"  OR  "hormonal replacement therapy"  OR  "estrogen"  OR  "hormones"  OR  "postmenopausal" )  AND  "Carpal Tunnel Syndrome" ) |
| Cochrane | ( ( "Estrogen Replacement Therapy"  OR  "hormonal replacement therapy"  OR  "estrogen"  OR  "hormones"  OR  "postmenopausal" )  AND  "Carpal Tunnel Syndrome" ) |
